# Challenges for Older Drivers in Urban, Suburban, and Rural Settings

**DOI:** 10.3390/geriatrics3020014

**Published:** 2018-03-22

**Authors:** Rashmi P. Payyanadan, John D. Lee, Lorelie C. Grepo

**Affiliations:** Department of Industrial and Systems Engineering, College of Engineering, University of Wisconsin-Madison, Madison, WI 53706, USA; john.d.lee@wisc.edu (J.D.L.); grepo@wisc.edu (L.C.G.)

**Keywords:** older driver, route choice, route preference, driving challenges, driver support systems, in-vehicle technologies

## Abstract

Along with age-related factors, geographical settings—urban, suburban, and rural areas—also contribute to the differences in fatal crashes among older drivers. These differences in crash outcomes might be attributed to the various driving challenges faced by older drivers residing in different locations. To understand these challenges from the perspective of the older driver, a focus group study was conducted with drivers 65 and older from urban, suburban, and rural settings. Guided-group interviews were used to assess driving challenges, mobility options, opportunities for driver support systems (DSS), and alternate transportation needs. Content analysis of the interview responses resulted in four categories representing common challenges faced by older drivers across the settings: behavior of other drivers on the road, placement of road signs, reduced visibility of road signs due to age-related decline, and difficulties using in-vehicle technologies. Six categories involved location-specific challenges such as heavy traffic situations for urban and suburban drivers, and multi-destination trips for rural drivers. Countermeasures implemented by older drivers to address these challenges primarily involved route selection and avoidance. Technological advances of DSS systems provide a unique opportunity to support the information needs for route selection and avoidance preferences of drivers. Using the content analysis results, a framework was built to determine additional and modified DSS features to meet the specific challenges of older drivers in urban, suburban, and rural settings. These findings suggest that there is heterogeneity in the driving challenges and preferences of older drivers based on their location. Consequently, DSS technologies and vehicle automation need to be tailored to not only meet the driving safety and mobility needs of older drivers as a population, but also to their driving environment.

## 1. Introduction

By 2060, the percentage of adults 65 years and older in the U.S. is expected to grow by 9 percent [1]. Along with a higher proportion of the population expected to be in the age group 65 and older, the number of licensed older drivers is also growing, increasing by 21 percent from 2002 to 2011, and accounting for 16 percent of all licensed drivers in the U.S. [2]. This shift towards more older drivers on the road has brought about a need to reassess their driving challenges, and related mobility and driving safety outcomes so as to develop a more comprehensive understanding and support structure to prolong their driving safety, mobility, and independence. 

In 2015, 6800 older adults in the U.S. were involved in fatal crashes and more than 260,000 were admitted to emergency departments for crash injuries [3]. Per mile traveled, drivers 70–74 years are more likely to be involved in a fatal crash, with the highest increase in crashes among those 85 years and older [4]. Numerous studies have highlighted the risk factors for increased motor vehicle crashes among older drivers due to age-related decline. Risk factors include cognitive impairments [5], reduced visual and motor function [6], and decline in physical functioning [7]. These age-related factors can degrade driving skills over time.

Older adults are generally safe drivers. But age-related decline can increase difficulty conducting certain driving maneuvers such as left turns and intersection negotiations [8]. When driving conditions become challenging—especially due to declining health, it can affect driving ability. Under such conditions, older drivers limit their driving through self-regulation by avoiding difficult driving situations such as rush hours, intersections, nighttime driving, unfamiliar areas, and bad weather [9]. Experiencing a motor vehicle crash also prompts self-regulation, such that drivers avoid driving in the rain and driving in rush hour [10]. Such self-regulation, although important for maintaining driving safety and prolonging mobility, it can often result in the decision to cease driving.

Driving cessation occurs when the driver, the family, or caregiver decides that driving becomes unsafe, impractical or impossible due to diminished health, functional capacity, or other circumstances of life [11]. Ross et al. [12] reported that the odds ratio of driving cessation for older adults to be 1.11 for each additional year of age and 1.15 for each additional medical condition. Other studies have found that common reasons for driving cessation among older adults were health problems, loss of confidence, and giving up driving on advice from family, friends, or a medical specialist [13,14]. These findings show that driving cessation mainly reflects safety concerns.

Driving cessation can have a huge cost on personal well-being and quality of life, and reduce participation in social and leisure activities among older adults [15]. The cost of cessation can be assessed from a monetary, time, and loss of opportunity perspective [16]. Driving cessation has been shown to increase depressive symptoms among older adults compared to those older adults who continue to drive [17,18]. And older adults who stopped driving were three times more likely to use mental health care services than current drivers [19]. Additionally, reduced driving exposure or driving cessation showed an 8 percent decrease in social engagement activities [20], and out-of-home activities compared to active older drivers [21]. Reduced activity also increases burden and reliance on family and friends: older adults who have stopped driving depend solely on their spouses and children for medical trips [20]. Thus, driving cessation can incur large personal, social, and community costs among older adults.

To reduce the costs of driving cessation, several studies have considered how to mitigate the driving challenges and concerns for older drivers. Loss of independence and lack of alternative transportation options are some of the reasons older drivers prefer to continue driving. But frequent near misses, inability to self-regulate, loss of confidence in their driving abilities, and declining health are some of the barriers to continued driving [22,23]. Although many studies have been conducted to understand the driving challenges faced by older drivers under different driving situations, few have explored these challenges across different geographical settings—urban, suburban, and rural.

According to the U.S. Census Bureau, urban is defined as a territory, population or housing units with a population density of 500–1000 people per square mile within an urbanized area or cluster. Suburban areas, also outside urbanized areas, have less access to resources and have a larger population than rural areas. Whereas rural is defined as areas that are outside the urbanized areas. Older adults comprise 12 percent of the population living in urban settings and 16 percent in rural settings. Among the 47 million people living in rural areas in the U.S., 7.5 million are above the age of 65 [24]. For older drivers, 61 percent of fatal crashes occur on rural roadways compared to urban roadways [25]. Using FARS and GES data (https://www-fars.nhtsa.dot.gov/main/index.aspx), Zwerling et al. [26] found that the fatal crash incidence density for older drivers was two times higher on rural roads compared to urban roads, where fatal crash incidence reflects both the risk of crashing and driving exposure. Although the majority of fatal crashes occurred on rural roads, both urban and suburban settings contributed to different types of crashes. In urban settings, older drivers were increasingly likely to be involved in single- and two-vehicle crashes, attributed to the greater number of intersections in urban areas [25]. Whereas in suburban settings, older drivers were more likely to be involved in crashes on two-lane roadways and multi-lane roads with speed limits of 40–45 mph. These results suggest that different geographical areas might raise different driving safety concerns. While much of the research considering the effect of different geographical areas on driving challenges has been conducted using crash data and self-reports, few have considered the challenges from the perspective of the older driver in these settings.

One of the few studies from the perspective of the older driver used a Contextual Inquiry approach to show that urban older drivers had more safety concerns related to traffic situations, and other drivers on the road not adhering to the rules but were less fearful of driving cessation due to the availability of alternate transportation options; whereas, rural older drivers were challenged by poor road infrastructure, and feared driving cessation due to lack of alternate transportation options [27]. Johnson [28] conducted a questionnaire study that also included semi-structured interviews to understand the decision to cease driving among rural older drivers. The study found that accidents, insecurity, impaired health, and social support influenced the decision to cease driving. The Transportation and Older Persons: Perceptions and Preferences report [29] showed that although driving a personal vehicle, riding with friends and family, and knowledge of transportation resources were important factors for improving the mobility of older drivers, rural older drivers were excluded due the greater challenges faced by rural drivers for these categories. Overall, studies conducted to understand the mobility needs of older drivers have covered topics related to driving cessation, crash risk, perception and attitudes toward different modes of transportation, and the importance of driving [30]. But few have delineated the differences in challenges that occur within the different geographical settings, which can provide insights for personalizing driver support systems (DSS) to meet these challenges.

The goal of this study is to use focus group interviews to understand the driving challenges of older drivers across three geographical locations—urban, suburban, and rural settings. Content Analyses—a method for analyzing written, verbal, or visual data, and making inferences from the data to their context with the goal of providing knowledge and new insights [31], was used to analyze the focus group responses. Results from the content analysis were used to identify factors within each geographical location that older drivers found challenging. These factors were then used to develop a framework for personalizing DSS technologies to provide more targeted interventions to improve and prolong the driving safety and mobility needs of older drivers.

## 2. Methods

Three focus groups were conducted with drivers 65 years and older living in urban, suburban, and rural settings in a Midwestern state in the United States. Each focus group received a demographic questionnaire to fill out before the focus group session. At each focus group session, a guided-group interview process with the help of a moderator was conducted. Participants were asked to respond to a series of open-ended questions related to the driving challenges faced in their geographical location, barriers to mobility, and interventions to aid driving.

### 2.1. Recruitment

Recruitment was conducted by contacting local senior centers that represented urban, suburban, and rural counties in a Midwestern state in the U.S. Flyers were sent to senior centers to post on their bulletin boards. The research team also advertised through the local radio news.

### 2.2. Participants

A total of 34 adults 65 years and older from urban, suburban, and rural settings were recruited for the focus group study. From each of the study participants, demographic data were collected before the session. Older adults participating in the focus group study were required to hold a valid driver’s license.

### 2.3. Focus Groups

A focus group approach was used in this study to understand the driving challenges of older adults in urban, suburban, and rural settings. These driving challenges reflect differences in complex behaviors and motivations [32], which are not reflected in crash data and are not easy to extract from controlled experiments or surveys. The focus group approach is a particularly powerful means of uncovering complex and highly contextualized behaviors because it benefits from the *group effect* [33]. The *group effect* is an outcome of interactions between individuals in focus groups that helps better understand assertions made by individuals in the group, wherein individuals query and explain themselves to each other [33].

Three focus group sessions were conducted, one in each of the urban, suburban, and rural settings. Each focus group session was 90 min long and involved 10–12 older drivers per group. For each focus group session, audio and video recording equipment were installed to record the session. Transcripts were made of the recorded sessions.

The purpose of the focus group sessions was to understand the location-specific driving challenges faced by older drivers. A moderator was present at each session to guide the discussion and ensure participation of all attendees. To understand the driving challenges faced by older drivers, the moderator guided the participants to respond to location-specific driving challenges related to their mobility barriers, driving concerns, access to resources, alternate transportation needs, and opportunities for driver support systems to aid driving.

### 2.4. Content Analysis

**Figure 1:**
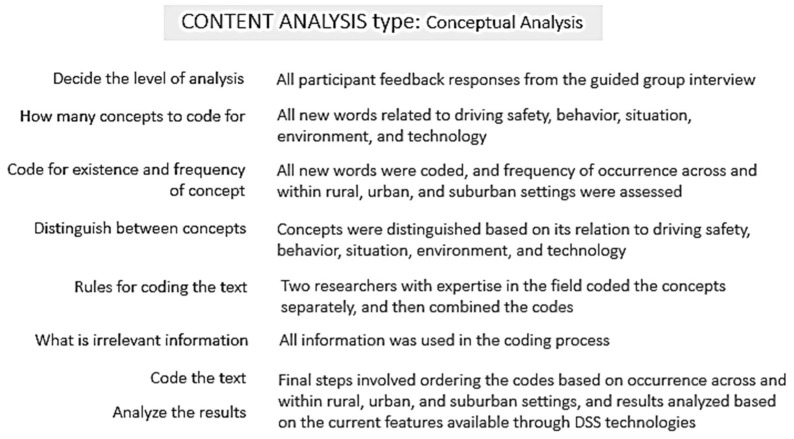
Steps for conducting the content analysis using the participant feedback responses.

Content analysis is a qualitative research method used for analyzing text data, identifying themes and patterns within the text data, coding through a systematic classification process, interpreting content and concepts, and assessing contextual meaning of the identified concepts [34]. Conceptual analysis, a type of content analysis was used to establish the existence and frequency of the concepts [35]. Responses from the participants were transcribed and were used to develop the major categories of driving challenges for older drivers as shown in Figure 1. These categories were used to highlight the limitations of driver support systems in meeting the driving challenges of older drivers at a location-specific level.

To develop the major categories of driving challenges, open coding was conducted to obtain all new words related to driving safety, driving behaviors, situations, driving environment, and technology. Once the coding reached saturation—where no more new codes emerged, axial coding followed. Axial coding categorized the codes into concepts. Spreadsheets were used to conduct open and axial coding. Two researchers conducted the open and axial coding. The inter-rater reliability for the coding phase was 0.814. The concepts that emerged from the content analysis process and their frequency of occurrence guided the development of a framework for describing the opportunities for DSS technologies to address the current driving challenges of older drivers at a location-specific level.

## 3. Findings

Results from the content analysis revealed four driving challenges that were common across urban, suburban, and rural settings, an additional six challenges that were location-specific. Challenges were related to driving, alternate transportation options, and issues practicing driving safety. Using the results from the content analysis, driver support systems (DSS) were identified with features that could address some of the driving challenges faced by older drivers. These results and current literature on DSS technologies were used to develop a framework to highlight the limitations in assessing the usefulness of DSS technologies for meeting the location-specific driver challenges of older drivers.

### 3.1. Older Drivers in Urban, Suburban, and Rural Settings

Table 1 summarizes the demographic data collected from the 34 adults 65 years and older who participated in the study. Compared to older adults in urban and suburban settings, older adults in rural settings drove 66 percent and 80 percent more miles/week, respectively. Suburban and rural participants were older than those from urban settings.

**Figure 2:**
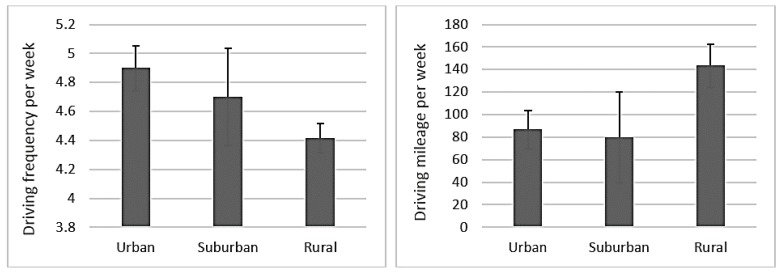
Reported driving frequency per week and driving mileage per week across urban, suburban, and rural participants.

Figure 2 shows the driving frequency per week and driving mileage per week reported by participants in the study across urban, suburban, and rural settings. The driving frequency per week of older drivers in each setting showed that there were no meaningful differences between their driving frequencies. Whereas driving mileage per week showed meaningful differences, which is likely a reflection of destinations in suburban and urban settings being closer in distance than rural settings.

### 3.2. Common Driving Challenges by Setting

Four driving challenge categories were common across urban, suburban, and rural areas—behavior of other drivers on the road, placement of road signs, reduced visibility of road signs due to age-related decline, and difficulty using in-vehicle technologies (Table 2). Aggressiveness and inattention of other drivers such as those using their cell phones while driving made older drivers feel unsafe. Older adults also reported a number of safety concerns with road signage: lack of standard sign placement, small street signs and illegible lettering, obstruction of signs due to trees, placement of signs not always on the near side, and lack of alerts to important signs ahead. And lastly, older drivers considered in-vehicle technologies to be a useful intervention for driving safety but had concerns with: limited experience using navigation systems, technology being too complicated, and the technology providing excessive alerts while driving.

To address these concerns, older drivers compensated through countermeasures. Some of the common countermeasures taken by older drivers to avoid these challenges depended on the driving situation and context. Table 2 shows the different strategies implemented by older drivers to reduce their driving challenges (ordered based on the most frequently stated responses), and their recommendations for improving driving safety.

### 3.3. Location-Specific Driving Challenges

Table 3 shows the six categories of driving challenges specific to the location, ordered by the most frequent responses. Driving through heavy traffic was a challenge mainly for older drivers in urban and suburban areas. Urban and suburban older drivers were also more likely to report having issues with alternate route choices when primary routes could not be taken. Reasons for considering alternate routes were primarily based on avoiding difficult driving maneuvers during medium to high traffic such as conducting left turns and roundabouts, construction zones, and detours. Urban older drivers were the only group to highlight concerns related to the lack of knowledge of certain driving rules, such as whether to slow down or speed up at a yellow light, and issues related to remembering and paying attention to the differing speed limits on certain roads. Urban older drivers also preferred using interstate highways because they reduced driving challenges—as there were fewer stops, fewer road signs, and less risky driving from others. 

In addition to these location-specific driving challenges, other factors also indirectly influenced driving challenges. These reflect the culture and transportation planning of the location, such as the lack of accessible public transportation options and economic costs. Urban and rural older drivers had issues related to accessibility of public transportation. In urban areas, older adults reported that public transportation was fragmented and hence did not provide adequate access to all areas. Whereas older adults in rural areas found long wait times and limited access a challenge, which deterred the use of public transportation. Lastly, for rural older drivers, economic concerns related to the cost of gas and tolls for long distance trips influenced their choice of route. To reduce economic costs, number of trips taken, and cost of gas, rural older drivers preferred to make multiple stops per trip. A major safety concern with rural roads was the lack of shoulders, which prevented older drivers from being able to safely pull over during an emergency, or due to poor visibility. Lack of turn lanes, unpaved and narrow roads also challenge older drivers in rural areas.

## 4. Can Driver Support Systems Help Address Older Driver Challenges?

Findings from the content analysis showed that the strategies implemented by older drivers in urban, suburban, and rural settings to reduce their driving challenges, and recommendations to help address these challenges, primarily involved route selection and avoidance. Technological advances of real-time routing applications provide driver support systems (DSS) with the unique capability to support the information needs for route selection and avoidance for drivers. The following section highlights the available DSS technologies that can address, to some extent, the specific driving challenges of older drivers (highlighted in Table 2 and Table 3), and assist in their route selection and avoidance. This is followed by an understanding of the limits of current DSS technologies, which merit further research and improvement to help address the driving safety and mobility needs of older drivers.

### 4.1. Current DSS Technology to Address Challenging Driving Situations

**Figure 3:**
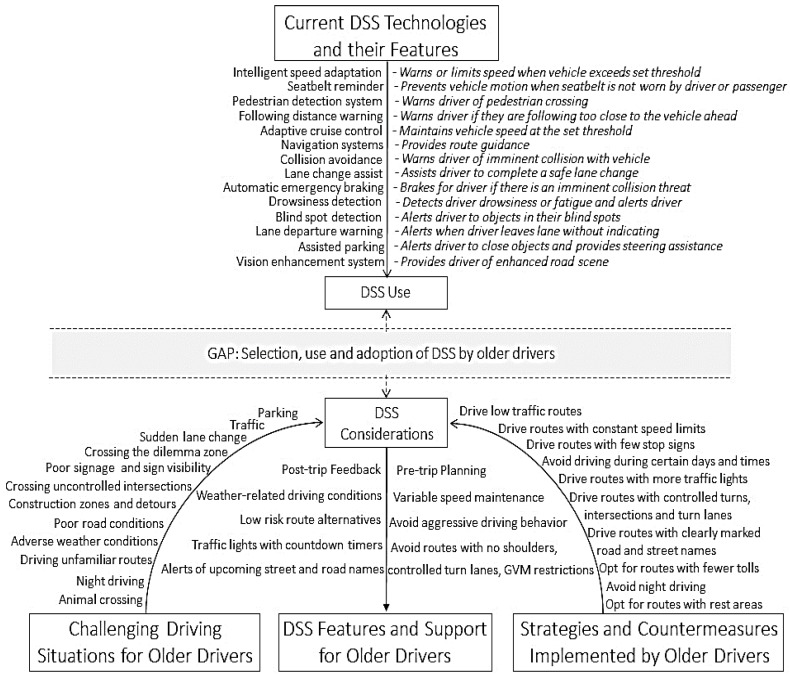
Framework for highlighting the current driver support systems (DSS) technologies that can help address the driving safety challenges of older drivers.

Figure 3 shows a framework that summarizes the current DSS technologies and their features: driving challenges of older drivers for each setting, strategies and countermeasures suggested by older drivers to address these challenges, and how DSS features could be tailored to address the challenges of older drivers in urban, suburban, and rural settings.

For each of the common and location-specific driving challenges, older drivers reported the following challenging maneuvers: parking, changing lanes, crossing uncontrolled intersections, driving through construction zones, on poor road conditions, under poor weather conditions, on unfamiliar roads, and night driving. Parking assistance systems such as Park Assist, Park Distance Control (PDC), Partial Automated Parking, and the Parking Garage Pilot enable automatic steering of the vehicle into parallel parking spaces, and in and out of tight parking spaces without the need for the driver to maneuver [36,37]. For Park Assist systems, the driver only operates the acceleration and braking during parking, while the PDC system provides information of the distance from obstacles through alerts and visual feedback. Urban and suburban older drivers would find such systems advantageous when parking spaces are tight. Parking was not a major concern for rural older drivers due to low-density in rural areas.

From the content analysis, older drivers considered blind spot detection a useful technology to have in vehicles, especially in urban and suburban areas. DSS technologies such as Lane-Change Collision and Avoidance Systems, allow for automatic steering to avoid obstacles [38]. And automated lane following, merging, and lane change systems such as Lane-Change Collision Warning Systems can alert drivers to objects in the driver’s blind spot. Thus, such technologies would be particularly beneficial for urban and suburban driving.

Results from the content analysis also showed that in suburban settings, intersections were preferred as they provided older drivers with a better assessment of time remaining to cross the intersection. In rural settings, maneuvering across intersections during the winter season was problematic as snow banks along the pavements would hinder visibility. Dotzauer et al. [39] tested intersection assistance system on older drivers and found that it reduced crossing time, and increased allocation of attention to the center of the road. Collision warning systems such as the Collision Avoidance/Warning System, Intersection Collision Avoidance System, and Reverse Collision Warning systems can also be useful as they are designed to alert drivers if there is an imminent collision with vehicles ahead, at intersections, and with rear objects [40].

In rural settings, older drivers reported a major driving safety concern was driving at night, under poor weather conditions, and unexpected animal crossing. Vision enhancement systems such as Night-Vision Enhancement Systems can help older drivers who have difficulty driving at night, on poorly lit roads, or in unfamiliar areas, and improve visibility of roadside objects and animals [41]. Although there is considerable research on the difficulties older drivers face reading signs, more research needs to be conducted to: understand how best to display these signs on dashboards, auditorially, or as a heads-up display, and to prioritize these signs to reduce driver workload [42].

Maneuvering through heavy traffic was also challenging for older drivers, especially in urban and suburban settings. Particularly on urban and suburban roads, older drivers reported greater numbers of traffic signs, vehicles, pedestrians, and cyclists compared to rural roads, which resulted in very demanding traffic situations. To address these challenges, DSS technologies such as the Advanced Transportation Information Systems (ATIS), Navigation and Route Guidance systems, and Variable Message Signs can provide urban and suburban older drivers with dynamic route and traffic information, to anticipate and make adjustments to alternate routes during a trip based on traffic conditions [43].

Additionally, older drivers in urban and suburban settings reported getting caught in the dilemma zone when they were unaware of the time left to cross an intersection, or due to high approaching speed, or late brake reaction time. DSS technologies to help eliminate dilemma zones are still limited. Liu et al. [44] studied the impact of increasing the yellow phase time to eliminate dilemma zones. Results showed that extending the yellow phase at intersections by 6 s eliminated dilemma zones for conservative drivers and reduced dilemma zones for normal and aggressive drives.

And lastly, older drivers reported preference for routes with less traffic, constant speed limits, visible road sign and placement, and controlled intersections. DSS technologies such as Curve Management system and Curve Speed Assistance help reduce driver’s speed when they approach a curve [45]. For roads with varying speed limits, especially in urban settings, Legal Speed Limit Assistance provide warnings or alerts when the vehicle speed exceeds the posted speed limit [46]. To assist drivers with turn-taking for left turns, U-turns, roundabouts, and crossing intersections, communication capabilities with roadside infrastructure can enhance the capabilities of DSS technologies by gathering information about the road environments. Trip planning and route selection systems could also help drivers select routes with less traffic, consistent speeds, and controlled intersections.

### 4.2. Future Work for DSS Technologies to Help Address Challenging Driving Situations

Based on the challenging driving situations and strategies implemented by older drivers, current DSS technologies are limited in addressing their concerns. *Pre-trip planning* is a useful DSS feature that is part of navigational systems. But current pre-trip planning features have not been adapted to fit the needs of older drivers to provide feedback based on their specific driving challenges and preferences. Navigation systems can be adapted to enable pre-trip planning features suited for older drivers by providing choice of low traffic routes, routes with fewer turns, optimal time of departure to avoid hazardous driving situations such as traffic congestion and poor weather conditions, routes that are familiar, avoid routes with tolls, highways, construction zones, and routes with no GVM restrictions. Payyanadan, Sanchez, and Lee [47] showed that there is opportunity to improve the driving safety outcomes of older drivers in urban and rural settings by providing them with alternate routes that reduce their exposure to left turns, U-turns, construction zones, and lane closures. Current DSS are not equipped with retrospective feedback features to provide *post-drive feedback* that can remind older drivers of the route driven and their driving behavior, challenges experienced along a driven route such as getting lost or missing a turn, and opportunities to improve driving safety outcomes by selecting alternate routes with fewer challenges. Several studies have shown that providing older drivers with retrospective feedback can provide older drivers with safety-critical feedback about their trips, and reduce their risky driving behaviors and route risk [48,49].

DSS technologies can be also be updated to provide information in advance about the *driving environment and weather conditions* such as information about the upcoming street and road names, changes in speed limits, and weather updates that might affect visibility, traffic flow, and road conditions. Two studies have shown that messages from Driver Assistance Systems to inform older drivers about the right-of-way regulation, view of the intersection, and safe gaps for joining or crossing traffic can result in safer driving performance [50]. Whereas the ADAS Horizon Provider [51] can present information ahead of the vehicle and provide drivers with the opportunity to anticipate and plan their route. To provide such information features especially to older drivers, DSS technologies also need to safely direct driver attention to unexpected changes in their driving environment and not require them to change their driving behavior suddenly from routine to planned behavior, enable the option to choose information delivery—either through speech or visual feedback, provide timed information that allows additional time to respond and plan for the driving situation, organize information based on the corresponding spatial and temporal structures of the driving environment, and prioritize information and alerts such that early warning is provided for unfamiliar situations.

### 4.3. Limitations in the Selection, Adoption, and Use of Current DSS Technologies by Older Drivers

Despite the opportunity for DSS technologies to improve driving safety for older drivers, there are a number of issues that deter the selection, adoption, and use of these technologies. Limited studies have shown that for navigational systems, different results have been reported on the benefits of DSS technologies for older drivers. Work by Dingus et al. [52] showed that older drivers using DSS technologies had difficulty driving and navigating concurrently, and had more safety-related errors compared to younger drivers. Results from the European DRIVE II Project, EDDIT showed that older drivers using DSS technologies were more confident when driving in unfamiliar and congested areas, and the system did not affect their driving safety or attention. But DSS technologies need to be adapted to fit the location-specific needs of older drivers especially because there is a lot of information to process in a short period of time, particularly in suburban areas with higher traffic [53]. In rural areas, the use of DSS systems is often considered unreliable as rural side roads are not always updated on DSS systems.

Additional limitations due to increased driving workload can hinder the adoption and use of DSS technologies among older drivers [54]. Older adults as a cohort have been reported to have a lower assessment of their skills and abilities for using and learning technologies compared to other age groups [55]; and their technology use often depends on the availability of training [56], as well as the trade-off between desired utility and perceived difficulty learning [54]. Lastly, there is overall very limited work in understanding how improved features of these technologies benefit older drivers as a cohort. Thus the selection, adoption, and use of DSS technologies that can reduce the driving challenges and inform the route choice preferences for older adults should take these factors into consideration.

Although current DSS technologies such as adaptive cruise control and forward collision warning/avoidance systems can help drivers regulate and maintain their speed, modelling these technology features to learn and reflect the risk-averse driving characteristics of older drivers such as maintaining greater headway distance based on the vehicle ahead, or providing alerts of vehicles displaying poor driving behavior can be a useful feature. Current research in automation has increased the focus on developing algorithms that predict driving style, and enable different driving style modes based on the driver’s preference behind the wheel [57]. And lastly, more work needs to be done to address the age differences in driving challenges within the older adult population, as rural drivers tend to be older than urban and suburban older drivers [58].

The design and implementation of DSS technologies has made significant progress over the past 15 years. A large body of research has been conducted to test their effects on road safety, behavioral adaptation, and design, albeit cohort-specific studies. Results of the safety benefits analyses of DSS technologies have shown promising results. Intelligent braking and lateral driver support systems have a safety potential of 40.8 percent of all car accidents avoided for Collision Mitigation Braking Systems (CMBS), 16.8 percent for Lane Keeping Assist Systems, 1.4 percent for Blind Spot Detection systems, and 24.7 percent for Lane Change Assist systems [59]. A more recent report on the estimated effectiveness in improving safety outcomes with DSS technologies showed an 81 percent reduction in backup collisions for Reverse Collision Warning (RCW) systems, 57 percent reduction in rear-impact crashes for Following Distance Warning systems, 50 percent reduction in serious injury and fatal rear-impact crashes for Adaptive Cruise Control systems, and 48 percent crashes avoidance with pedestrians for Pedestrian Detection systems [40]. Based on these trends and insights on the driving challenges and countermeasures adopted by older drivers, there is opportunity for DSS technologies and future work in automation to reduce the driving challenges as well as improve safety outcomes for older drivers.

The current study has a number of limitations that need to be highlighted. The study included a single focus group from each location, and so represents a small sample of drivers from each location. Thus, findings from this study should be extrapolated with caution. The small sample size also limited the scope of the analyses: the content analysis could be used to determine concepts rather than trends [34]. Although the focus group approach provided ideas and considerations for improving the driving challenges of older drivers, the comparability and generalizability of the challenges across groups also needs to be extrapolated with caution. Additionally, a drawback of the focus group approach is the potential confounding influence of interactions with other participants, such as how pressures of social conformance might influence the discussion and resulting data [60]. This limitation stems from the benefit of social interactions surfacing attitudes, and explanations that individual interviews and observations may not reveal, and so represents a tradeoff associated with methods used to understand driver behavior. Such tradeoffs should be addressed with future work to further our understanding of the challenges of older drivers using naturalistic driving data, which provides an objective representation of the driving challenges faced by older drivers in urban, suburban, and rural settings [47].

## 5. Conclusions

Although current DSS technologies are built to improve the driving safety outcomes of the general driving population, they are not always designed with older drivers in mind. The content analysis revealed that older drivers in the study had different driving challenges based on their geographical location, with route selection and avoidance as the primary strategy implemented to overcome these challenges. While many of the current DSS technologies address the challenges related to driving maneuvers and traffic, features of these technologies related to route selection and avoidance are still in its nascent stages and provide limited options to address the route information needs of older drivers. Pre-trip planning and retrospective feedback on route alternatives are features that could improve the driving safety outcomes of older drivers. Thus, for DSS technologies to improve the driving safety outcomes of older drivers, their implementation needs to consider various aspects of the aging process from individual preferences and challenges, to location-specific demands of the driving environment. Better understanding how DSS technologies can be tailored to fit the needs of older drivers can enhance their safety, as well as enhance their mobility and prolong their period of independent living.

## Figures and Tables

**Table 1:** Demographic data of older drivers by location.

Settings	Participants	Age	Miles Driven/Week
Urban	12	71.9 (1.2)	86.75 (19.0)
Suburban	10	75.8 (2.3)	79.75 (17.1)
Rural	12	77 (2.3)	143.75 (40.2)

**Table 2:** Common driving challenges of older drivers and the strategies implemented to overcome their driving challenges in urban, suburban, and rural settings.

Common Driving Challenges across Urban, Suburban, and Rural Settings	Characteristics of the Driving Challenge and Examples of the Responses from Older Drivers Regarding These Challenges	Countermeasures Taken by Older Drivers and Their Recommendations to Help Avoid These Challenges
Driving behavior of other drivers on the road	Driving challenges due to the behavior of other drivers on the road such as inattentiveness and aggressive driving. *‘Other drivers not paying attention’* *‘Too much cell phone use while driving’*	Avoid certain routes *‘Freeway has too many aggressive drivers so I avoid it’* Defensive driving *‘We practice defensive driving to avoid hitting other drivers’* *‘To deal with other drivers poor behavior, I keep a more than safe distance and try to predict how they are driving’*
Placement of road signs	Challenges related to the legibility and placement of road signs. *‘The road signs are so inconsistent and you can’t read…many are faded’* *‘There are so many signs it gets confusing and then I wind up in the wrong place or taking a wrong turn’* *‘The road signs are bad enough that sometimes I can’t find an address because I feel like the signs are wrong’*	Require consistent sign placement *‘It would be good to have road signs more evenly spaced, larger, and maybe some reminder signs to remind us of what we read a mile ago’* Require better reflective signage *‘The signs are harder to read at night. I have to squint. Wish they were larger but also had reflection so that they are easier to read in the headlights’*
Reduced visibility of road signs due to age-related decline	Age-related vision limitations of drivers that result in diminished depth perception and challenges reading road markings and signs, especially at night, and under poor weather conditions. *‘When the weather is bad I can forget about being able to read signs at night. Can’t see any of it’* *‘My doctor said I have poor depth perception so it is even harder for me to read anything at night*	Avoid night driving *‘I hate driving at night because there are no proper lit roads for the entire journey and I would hate to deal with hitting an animal’* Defensive driving *‘We just avoid anything we aren’t comfortable with: bad roads, night driving, unfamiliar areas’*
In-vehicle technology use	Issues using driver support systems such as complexity of the technology (too much information to process), uncertainty about the recommended routes, and inaudibility of audio feedback from navigational systems. *‘I don’t want all of that coming at me’* (in reference to in-vehicle warnings) *‘Because GPS information is usually hard to hear, it’s good to have a co-pilot’*	In-vehicle technologies applications should be simple to use and understand *‘Any technology should assume that we will not read instructions’* In-vehicle technologies should have weather updates *‘Weather conditions should be part of the GPS systems when we look at alternative routes to take’*

**Table 3:** Location-specific driving challenges of older drivers and the strategies implemented to overcome their driving challenges.

Driving Challenges	Settings	Characteristics of the Driving Challenge and Examples of the Responses from Older Drivers Regarding These Challenges	Strategies Implemented by Older Drivers and Recommendations to Help Avoid These Challenges in Specific Settings
Heavy traffic conditions	Urban Suburban	Heavy traffic refers to the volume of vehicles (congestion) and the types of vehicles (e.g., big trucks) on the road that make drivers feel unsafe. *‘Too many drivers on the roads’*	Avoid rush hours and certain days of the week *‘I avoid Fridays because there are more drivers on the roads in advance of the weekend, and the first day of each month because of payday’* Avoid roads where big trucks frequent *‘Big trucks are intimidating on interstates so I avoid them’* Avoid roads based on traffic conditions *‘Use of side or residential roads is helpful for avoiding the busy, cluttered streets’*
Unclear driving rules	Urban	Challenges related to lack of knowledge of the particular road rules or conduct a driving maneuver safely. *‘I really don’t know whether I should proceed through the light or stop when I see the yellow light’*	Drive routes with fewer rules *‘I prefer interstate driving in town because people follow the rules more, and more predictable driving behaviors, and because less information to look out for’*
Difficult driving maneuvers on certain roads	Urban Suburban	Challenges related to specific driving maneuvers that are difficult on certain roads *‘Roundabouts both helpful and dangerous. More dangerous at busy intersection, especially at interstate on-ramps/off-ramps than in residential areas’* *‘Construction zones difficult to drive through’*	Drive familiar routes *‘To help with left turns, will go to known intersections with traffic lights that have left-turn signals’* Opt for alternate routes depending on available route options *‘Alternate routes sometimes not possible because routes limited’*
Animal crossing	Rural Suburban	Crash risk and near-miss concerns due to animal crossing and farm equipment. *‘Hitting deer is a common occurrence’* *‘Uncertain events on rural roads like blind driveways, farm equipment, deer’*	Avoid night driving *‘Avoid driving at night due to poor visibility, especially because unable to see deer and other hazards such as buggies’*
Public transportation	Urban Rural	Challenges with alternate transportation options such as limited accessibility and long wait times. *‘County offers a van service but long wait times’* *‘[Urban city] segmented into four main areas (N, S, W, E) that each have distinct driving environments and driver types; because of this, public transportation options limited and do not effectively cross these boundary lines’*	Driving is the primary and most convenient mode of transport *‘I don’t want to wait, I just want to go’*
Economic concerns	Rural	Gas prices and toll fees *‘Gas economy important consideration for planning trips’*	Avoid tolls *‘Go out of way to avoid tolls’* Plan routes and stops in advance *‘Trips planned for efficiency’* *‘Try to only make one trip in a day and plan multiple stops in a circular pattern’*

## References

[1] Colby S.L., Ortman J.M. (2014). Projections of the Size and Composition of the US Population: 2014 to 2060.

[2] National Highway Traffic Safety Administration (NHTSA) (2014). Traffic Safety Facts 2012 Data.

[3] Centers for Disease Control and Prevention (2017). Web-Based Injury Statistics Query and Reporting System (WISQARS).

[4] Insurance Institute for Highway Safety (IIHS) (2015). Fatality Facts 2015, Older People.

[5] Lundberg C., Hakamies-Blomqvist L., Almkvist O., Johansson K. (1998). Impairments of some cognitive functions are common in crash-involved older drivers. Accident Anal. Prev..

[6] Rubin G.S., Ng E.S.W., Bandeen-Roche K., Keyl P.M., Freeman E.E., West S.K., Alston C., Alston D., Donoway D., Harrison S. (2007). A prospective, population-based study of the role of visual impairment in motor vehicle crashes among older drivers: The SEE study. Investig. Ophthalmol. Vis. Sci..

[7] Marottoli R.A., Cooney L.M., Wagner D.R., Doucette J., Tinetti M.E. (1994). Predictors of automobile crashes and moving violations among elderly drivers. Ann. Intern. Med..

[8] McGwin G., Brown D.B. (1999). Characteristics of traffic crashes among young, middle-aged, and older drivers. Accident Anal. Prev..

[9] Okonkwo O.C., Crowe M., Wadley V.G., Ball K. (2008). Visual attention and self-regulation of driving among older adults. Int. Psychogeriatr..

[10] Ball K., Owsley C., Stalvey B., Roenker D.L., Sloane M.E., Graves M. (1998). Driving avoidance and functional impairment in older drivers. Accident Anal. Prev..

[11] Meuser T.M., Berg-Weger M., Chibnall J.T., Harmon A.C., Stowe J.D. (2013). Assessment of Readiness for Mobility Transition (ARMT): A tool for mobility transition counseling with older adults. J. Appl. Gerontol..

[12] Ross L.A., Anstey K.J., Kiely K.M., Windsor T.D., Byles J.E., Luszcz M.A., Mitchell P. (2009). Older drivers in Australia: Trends in driving status and cognitive and visual impairment. J. Am. Geriatr. Soc..

[13] Brayne C., Dufouil C., Ahmed A., Dening T.R., Chi L.Y., McGee M., Huppert F.A. (2000). Very old drivers: Findings from a population cohort of people aged 84 and over. Int. J. Epidemiol..

[14] Gilhotra J.S., Mitchell P., Ivers R., Cumming R.G. (2001). Impaired vision and other factors associated with driving cessation in the elderly: The Blue Mountains Eye Study. Clin. Exp. Ophthalmol..

[15] Herzog A.R., Ofstedal M.B., Wheeler L.M. (2002). Social engagement and its relationship to health. Clin. Geriatr. Med..

[16] Litman T. (2009). Transportation Cost and Benefit Analysis.

[17] Marottoli R.A., de Leon C.F.M., Glass T.A., Williams C.S., Cooney L.M., Berkman L.F., Tinetti M.E. (1997). Driving cessation and increased depressive symptoms: Prospective evidence from the New Haven EPESE. Established Populations for Epidemiologic Studies of the Elderly. J. Am. Geriatr. Soc..

[18] Windsor T.D., Anstey K.J., Butterworth P., Luszcz M.A., Andrews G.R. (2007). The role of perceived control in explaining depressive symptoms associated with driving cessation in a longitudinal study. Gerontologist.

[19] Naumann R.B., Dellinger A.M., Anderson M.L., Bonomi A.E., Rivara F.P. (2012). Healthcare utilization and costs among older adult female drivers and former drivers. J. Saf. Res..

[20] Taylor B.D., Tripodes S. (2001). The effects of driving cessation on the elderly with dementia and their caregivers. Accident Anal. Prev..

[21] Siren A., Hakamies-Blomqvist L. (2004). Private car as the grand equaliser? Demographic factors and mobility in Finnish men and women aged 65+. Transp. Res. Part F Traffic Psychol. Behav..

[22] Adler G., Rottunda S. (2006). Older adults’ perspectives on driving cessation. J. Aging Stud..

[23] Yassuda M.S., Wilson J.J., Mering O.V. (1997). Driving cessation: The perspective of senior drivers. Educ. Gerontol..

[24] Jones C.A. (2007). Population dynamics are changing the profile of rural areas. J. Rural Ment. Health.

[25] Stutts J., Martell C., Staplin L. (2009). Identifying Behaviors and Situations Associated with Increased Crash Risk for Older Drivers.

[26] Zwerling C., Peek-Asa C., Whitten P.S., Choi S.W., Sprince N.L., Jones M.P. (2005). Fatal motor vehicle crashes in rural and urban areas: Decomposing rates into contributing factors. Inj. Prev..

[27] Payyanadan R.P., Gibson M., Chiou E., Ghazizadeh M., Lee J.D. (2016). Contextual Design for Driving: Developing a Trip-planning Tool for Older Adults. Transp. Res. Part F Traffic Psychol. Behav..

[28] Johnson J.E. (1995). Rural elders and the decision to stop driving. J. Commun. Health Nurs..

[29] Coughlin J. (2001). Transportation and Older Persons: Perceptions and Preferences.

[30] Gardezi F., Wilson K.G., Man-Son-Hing M., Marshall S.C., Molnar F.J., Dobbs B.M., Tuokko H.A. (2006). Qualitative Research on Older Drivers. Clin. Gerontol..

[31] Krippendorff K. (1980). Validity in content analysis. Computerstrategien für die Kommunikationsanalyse.

[32] Morgan D.L., Krueger R.A. (1993). When to use focus groups and why. Successful Focus Groups: Advancing the State of the Art.

[33] Carey M.A., Smith M.W. (1994). Capturing the group effect in focus groups: A special concern in analysis. Qual. Health Res..

[34] Hsieh H.F., Shannon S.E. (2005). Three Approaches to Qualitative Content Analysis. Qual. Health Res..

[35] Busch C., de Maret S.P., Flynn T., Kellum R., Le S., Meyers B., Saunders M., White R., Palmquist M. (2012). Content Analysis. Writing@CSU. http://writing.colostate.edu/guides/guide.cfm?guideid=61.

[36] Taylor M.C., Lynam D.A., Baruya A. (2000). The Effect of Drivers’ Speed on the Frequency of Road Accidents.

[37] ERTRAC Task Force (2015). Automated Driving Roadmap.

[38] Regan M.A., Oxley J.A., Godley S.T., Tingvall C. (2001). Intelligent Transport Systems: Safety and Human Factors Issues.

[39] Dotzauer M., Caljouw S.R., de Waard D., Brouwer W.H. (2013). Intersection assistance: A safe solution for older drivers?. Accident Anal. Prev..

[40] Kweon Y.-J. (2011). Crash data sets and analysis. Handbook of Traffic Psychology.

[41] Mitchell C.G.B., Suen S.L. (1998). Urban Travel, Intelligent Transportation Systems, and the Safety of Elderly and Disabled Travelers. J. Urban Technol..

[42] Charles M., Haddad H. (2007). Prolonging the Safe Driving of Older People through Technology.

[43] Firmin P.E. Satellite Navigation Technology Applications for Intelligent Transport Systems: A European Perspective. Proceedings of the European Navigation Conference.

[44] Liu Y., Chang G., Tao R., Hicks T., Tabacek E. (2007). Empirical Observations of Dynamic Dilemma Zones at Signalized Intersections. Transp. Res. Rec..

[45] Anders L., Chen F. (2007). State of the art analysis: An overview of advanced driver assistance systems (ADAS) and possible human factors issues. Human Factors and Economic Aspects on Safety.

[46] Lu M., Wevers K., van der Heijden R. (2005). Technical Feasibility of Advanced Driver Assistance Systems (ADAS) for Road Traffic Safety. Transp. Plan. Technol..

[47] Payyanadan R.P., Sanchez F.A., Lee J.D. (2017). Assessing Route Choice to Mitigate Older Driver Risk. IEEE Trans. Intell. Transp. Syst..

[48] Payyanadan R.P., Maus A., Sanchez F., Lee J.D., Miossi L., Abera A., Melvin J., Wang X. (2017). Using trip diaries to mitigate route risk and risky driving behavior among older drivers. Accident Anal. Prev..

[49] Donmez B., Boyle L.N., Lee J.D. (2008). Mitigating driver distraction with retrospective and concurrent feedback. Accident Anal. Prev..

[50] Davidse R.J., Hagenzieker M.P., Wolffelaar P.C.V., Brouwer W.H. (2009). Effects of in-car support on mental workload and driving performance of older drivers. Hum. Factors.

[51] Blervaque V., Mezger K., Beuk L., Loewenau J. (2006). Adas horizon—How digital maps can contribute to road safety. Advanced Microsystems for Automotive Applications.

[52] Dingus T.A., Hulse M.C., Mollenhauer M.A., Fleischman R.N., McGehee D.V., Manakkal N. (1997). Effects of age, system experience, and navigation technique on driving with an advanced traveler information system. Hum. Factors.

[53] Entenmann V., Küting H.J. Safety Deficiencies of Elderly Drivers and Options Provided by Additional Active Safety—A Driver Centred View. Proceedings of the 7th World Congress on Intelligent Transportation Systems (ITS).

[54] Melenhorst A.S., Rogers W.A., Bouwhuis D.G. (2006). Older adults’ motivated choice for technological innovation: Evidence for benefit-driven selectivity. Psychol. Aging.

[55] Marquié J.C., Jourdan-Boddaert L., Huet N. (2002). Do older adults underestimate their actual computer knowledge?. Behav. Inf. Technol..

[56] Rogers W.A., Fisk A.D., Mead S.E., Walker N., Cabrera E.F. (1996). Training older adults to use automatic teller machines. Hum. Factors.

[57] Suzdaleva E., Nagy I. (2018). An online estimation of driving style using data-dependent pointer model. Transp. Res. Part C Emerg. Technol..

[58] Horswill M.S., Pachana N.A., Wood J., Marrington S.A., McWilliam J., McCullough C.M. (2009). A comparison of the hazard perception ability of matched groups of healthy drivers aged 35 to 55, 65 to 74, and 75 to 84 years. J. Int. Neuropsychol. Soc..

[59] Kuehn M., Hummel T., Bende J. Benefit Estimation of Advanced Driver Assistance Systems for Cars Derived. Proceedings of the 21st International Technical Conference on the Enhanced Safety of Vehicles (EVS).

[60] Morgan D. (1996). Focus Groups. Annu. Rev. Sociol..

